# Mother Load: Arsenic May Contribute to Gestational Diabetes

**DOI:** 10.1289/ehp.117-a310b

**Published:** 2009-07

**Authors:** Julia R. Barrett

**Affiliations:** **Julia R. Barrett**, MS, ELS, a Madison, Wisconsin–based science writer and editor, has written for *EHP* since 1996. She is a member of the National Association of Science Writers and the Board of Editors in the Life Sciences

Chronic exposure to arsenic—usually via drinking water contaminated with inorganic arsenic—has been associated with an increased risk of type 2 diabetes mellitus in countries around the world. New research shows that arsenic exposure may be an environmental risk **[*EHP*** **117:1059–1064; Ettinger et al.]**.

Arsenic may promote type 2 diabetes by increasing insulin resistance (inability to utilize insulin at the cellular level) and impairing insulin production. Insulin resistance is also a central feature of gestational diabetes, a potential complication during pregnancy that can lead to a 30–60% increased risk for the mother of developing lifelong diabetes, as well as impaired glucose tolerance, adverse birth outcomes, and obesity in her child.

The study was conducted near the Tar Creek Superfund site in Ottawa County, Oklahoma, whose residents include many Native Americans, a population already at elevated risk for type 2 diabetes. The area, once active in lead and zinc mining, has an above-average poverty rate compared with the rest of Oklahoma and the nation. Mine waste contaminated with assorted metals is still present and has been used to build roads, playgrounds, driveways, and house foundations. Moreover, 25% of drinking water samples tested in the area have naturally occuring arsenic levels exceeding the Environmental Protection Agency maximum contaminant level of 10 μg/L.

Total arsenic concentrations were measured in blood and hair samples collected at delivery from 532 women; blood was available from all women and hair from a subset of 179. Routine prenatal glucose tolerance tests conducted between weeks 24 and 28 of pregnancy yielded plasma glucose measurements, and questionnaires and medical record review provided data on sociodemographic characteristics, potential sources of arsenic exposure, and pregnancy history. Blood arsenic concentrations, a measure of biologically active arsenic, were between 0.2 and 24.1 μg/L, whereas hair arsenic concentrations, an indicator of cumulative exposure, were 1.1–724.4 ng/g. Blood glucose levels ranged from 40 to 284 mg/dL. At a cut-off value of > 140 mg/dL, 12% of the women were identified as having impaired glucose tolerance; a cut-off value of 130 mg/dL yielded a prevalence of more than 20%. A statistically significant relationship existed between each increasing quartile of blood arsenic exposure and impaired glucose tolerance after controlling for health and demographic factors. Depending on the glucose test cut-off value, women in the highest quartile of arsenic exposure were 2.4–2.8 times more likely to have impaired glucose tolerance than women in the lowest quartile of exposure.

These results suggest that chronic arsenic exposure may increase the risk of developing gestational diabetes. A better understanding of this and other factors through further research may identify modifiable risk factors this condition.

## Figures and Tables

**Figure:**
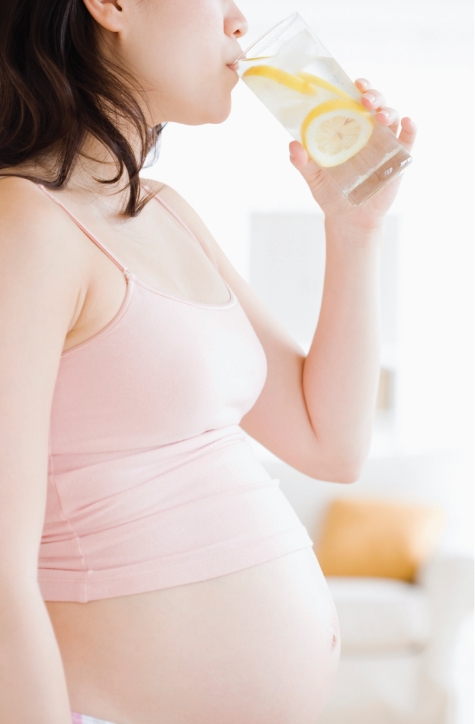
Millions of people worldwide may be exposed to naturally occurring arsenic in drinking water.

